# Prediction of Problematic Smartphone Use: A Machine Learning Approach

**DOI:** 10.3390/ijerph18126458

**Published:** 2021-06-15

**Authors:** Juyeong Lee, Woosung Kim

**Affiliations:** 1 Department of Industrial Engineering, Ulsan National Institute of Science and Technology, Ulsan 44919, Korea; passionlee428@unist.ac.kr; 2 College of Business Administration, Konkuk University, Seoul 05029, Korea

**Keywords:** smartphone addiction, problematic smartphone use, machine learning, predictor

## Abstract

While smartphone addiction is becoming a recent concern with the exponential increase in the number of smartphone users, it is difficult to predict problematic smartphone users based on the usage characteristics of individual smartphone users. This study aimed to explore the possibility of predicting smartphone addiction level with mobile phone log data. By Korea Internet and Security Agency (KISA), 29,712 respondents completed the Smartphone Addiction Scale developed in 2017. Integrating basic personal characteristics and smartphone usage information, the data were analyzed using machine learning techniques (decision tree, random forest, and Xgboost) in addition to hypothesis tests. In total, 27 variables were employed to predict smartphone addiction and the accuracy rate was the highest for the random forest (82.59%) model and the lowest for the decision tree model (74.56%). The results showed that users’ general information, such as age group, job classification, and sex did not contribute much to predicting their smartphone addiction level. The study can provide directions for future work on the detection of smartphone addiction with log-data, which suggests that more detailed smartphone’s log-data will enable more accurate results.

## 1. Introduction

With the rapid increase in smartphone penetration, they are becoming a part of our daily lives. Due to the various functions and convenience of smartphones, the number of users worldwide was more than 1.08 billion in early 2012, and continues to increase exponentially [1]. In the UK, 68% of adults were reported to own a smartphone, and the number of smartphone users in South Korea exceeded 39 million in early 2012 [2,3]. This trend is observed worldwide [4,5]. On the other hand, the number of people who depend too much on smartphones is also increasing. According to a survey of 29,712 smartphone users conducted by Korea Internet and Security Agency (KISA) in 2017, the high-risk smartphone addiction rate was about 18.6% (7860), which increased by about 1% since the previous year [6]. Further, the proportion of high-risk smartphone usage groups by age in the past two years increased from 6.7% to 19.1% among infants, the biggest increase among all age groups, followed by adults, with an increase from 3.9% to 17.4%. Among young adults and college students, the increasing reliance on smartphones has created a new potential for the widespread abuse of the technology in ways that suggest addiction. Many researchers call attention to the harmful effects of smartphone overuse, and various studies have been conducted (e.g., [7,8,9,10]). While various studies have focused on smartphone addiction, there is little research on the prediction of smartphone addiction based on usage pattern. Thus, the present study aimed to identify factors that predict the smartphone addiction level, such as smartphone usage level by contents, gender, age, and job. Accordingly, it attempted to develop a model to predict smartphone addiction level. For prediction, machine learning methods that are used commonly were tested.

Although smartphone addiction does not easily fit into the standard classification of impulse disorders according to the Diagnostic and Statistical Manual of Mental Disorders (DSM-5), the concept of smartphone addiction is increasingly becoming accepted [11]. The following problematic behaviors associated with smartphone use are most suggestive of addiction: (1) use in dangerous situations; (2) harm or repeated interruptions to work, social life, family life, and/or physical and mental well-being; (3) urges and drives to repeat behavior; (4) dependence, tolerance, and increasing need for stimulation to achieve satisfaction; and (5) anxiety or negative feelings associated with inability to send or receive immediate responses [12]. Similar symptoms have been reported in [13], and these have led some research to classify problematic smartphone use as a potential behavioral addiction [5,10,14]. Excessive use of smartphones can interfere with everyday life and can cause various physical, mental, and social problems [15]. A variety of activities can be conducted through one device, and most of the activities are classified into sedentary behavior, which is characterized by an energy expenditure of less than 1.5 metabolic equivalents (METs). Such behavior results in low levels of energy expenditure and correlates with health problems such as obesity or metabolic syndrome [16,17,18,19]. Excessive smartphone users showed less physical activity, such as a smaller number of steps taken per day, and they tended to consume fewer calories per day [20]. Other adverse physical effects include neck pain symptoms [21], craniocervical posture, dry eyes, carpal tunnel syndrome, sleep disturbance, and headaches [20,22,23]. Regarding mental health, smartphone overuse might be related to depression and anxiety [20,24]. Additionally, it can cause human relationship problems and reduce academic achievement [9,23,25]. A report published by KISA stated that “45.8% of smartphone users feel anxiety when they are not holding their smartphone, 27.1% spend too much time using their smartphone, and 22.6% have repeatedly attempted to reduce their smartphone use but have always failed. Moreover, 21% of smartphone users reported difficulties with school or work due to excessive smartphone use” [2,26]. Thus, smartphone use can become a serious problem because it involves the use of several addictive elements such as the internet and games [27]. Considering the importance of problems caused by excessive smartphone use, it is important for users to be aware of their condition.

In South Korea, the Smartphone Addiction Scale (S-scale) was developed by KISA to assess the current level of addiction [28]. The questionnaire consists of 10 questions to identify the level of smartphone addiction risk and to distinguish the high-risk group. While it provides useful information about the user’s current level of addiction, the questionnaire does not provide information on smartphone usage patterns, since it aims to diagnose the user’s psychological factors. Thus, even if classified as over-dependent by the S-scale, information about usage patterns cannot be obtained, and practical guidelines on addressing addiction problems cannot be developed. To identify the predictors of smartphone addiction, the data should be analyzed with multiple variables in an integrated manner. Although some mobile applications capture log data from smartphones, they have not been expanded to determine whether users are over-dependent [29]. Our research is motivated by the lack of results and our goal is to identify the predictors of smartphone addiction.

In this paper, we propose a model for predicting age-based smartphone addiction level using information available from smartphones, by exploring user groups that exhibit similar usage patterns. Our research is based on a survey called “A Survey on Smartphone Over-Reliance (SSOR)”. The survey is conducted by KISA every year and it comprises about 180 questions. The data are collected from 29,712 participants (14,790 male and 14,922 female) in 2017. The survey and the results of the respondents’ Smartphone addiction scale survey were integrated and analyzed. While our study relies on survey data (not actual usage information), a sufficient sample size was obtained. First, we conducted hypothesis tests to find out whether there is a difference in smartphone usage patterns and monthly spending between the normal user group and the problematic user group. The goal of the hypothesis test is to test whether there is a difference in the content used by the two groups of users. Except for the use of office search, web document and sports betting, there were significant differences in the usage level of all contents. In addition, significant differences were observed in the average monthly expenditure on games, movies/videos, and e-books and cartoons between two groups. In particular, those in the problematic group spent 2, 1.64, and 2.4 times more money on games, movies/videos, and e-books and cartoons, respectively, as compared to those in the normal group. As such, through the t-test, it is possible to figure out how different the usage patterns and expenditures are between the two groups. In addition to hypothesis testing, machine learning techniques were also used to reveal the relationship between usage pattern and smartphone addiction level. The samples were randomly divided into a training set consisting of 70% of all observations and a test set consisting of the remaining 30% of observations. Three learning methods were considered and tested: random forest, Xgboost and decision tree. The average values for accuracy were 82.59% (random forest), 80.77% (Xgboost) and 74.56% (decision tree), respectively.

The following can be derived from our results. First, the results show that information on users’ smartphone usage patterns and expenditure can be used as predictors to determine whether users are addicted to smartphones. Existing questionnaires developed to determine smartphone addiction are mostly focused on the psychological factors of users. Because information on usage patterns provides more objective information, the results of this study can be used to improve existing surveys. Second, the results may serve as evidence of high complexity in the smartphone addiction diagnosis. In our model based on learning techniques, the user’s personal information, content usage patterns and expenditure are required to ensure the sufficient accuracy. This means that various factors (age or content use) together influence the determination of smartphone addiction. Third, our results provide a basis for developing programs such as self-diagnostic applications to detect smartphone addiction. Note that our model uses only the information stored in the smartphone as predictors. If we can diagnose the smartphone addiction level just from the information stored in a smartphone (without psychological factors), this can provide useful information to users through the form of applications or programs. While the limitation of our research is that we rely on survey data (not actual usage information). We hope that the study can provide directions for future work on the detection of smartphone addiction with inputs, which suggests that more detailed smartphone’s log-data will enable more accurate results.

The remainder of the paper is organized as follows. In Section 2, we describe our predictive model and the data we used. In Section 3, we present our results. Discussions are presented in Section 4. Conclusions and future research directions are introduced in Section 5.

## 2. Methods

### 2.1. Participant

We collected data from 29,712 participants (14,790 male and 14,922 female) who responded to a survey conducted by KISA in 2017. Participants were native Koreans aged from 3 years to those in their 60s, residing in metropolitan areas in South Korea. This survey comprised nine questions of the S-scale, which is used to diagnose respondents’ addiction level. Additionally, it comprised questions on basic personal information such as employment status, monthly income, and 180 questions about smartphone usage. These 180 questions pertained to aspects such as usage frequency on weekdays and weekends, and self-evaluation of usage level for each type of smartphone content. All questions were in the multiple-choice format based on predefined categories. Therefore, one can check the usage of smartphones according to each type of over-dependence.

Though the KISA data comprised 180 questions, 27 questions were employed for the smartphone addiction prediction model since the only answers for the 27 questions are available from smartphone log data. To strengthen the performance of the predictive model, initially, we divided respondents into the following seven age groups: 3–9 years, 10s, 20s, 30s, 40s, 50s, and 60s. Further, among them, only the respondents in their 10s, 20s, and 30s were selected as target respondents for the predictive model because the KISA survey was most interested in those aged below 40 years. According to the survey, addiction level was classified into the following three groups: high risk, potential risk, and normal. For the present study, those categorized as “potential risk” were assigned to the normal smartphone user group because we did not observe significant differences in the smartphone use patterns of this group as compared to normal usage.

To assess consistency of responses in each age group, we computed the Cronbach’s alpha, which represents the internal consistency of the survey or scale, expressed as a number between 0 and 1. The Cronbach’s alpha can be calculated as follows:

(1) $$\alpha =\frac{K}{K-1}\left(1-\frac{\sum_{i=1}^{K}\sigma_{Y_{i}}^{2}}{\sigma_{X}^{2}}\right),$$

where *K* is the number of questions, *Y_i_* is the answer for the *i*-th question and *X* is $\sum_{i=1}^{K}Y_{i}$.

Internal consistency indicates the extent to which all items in the test measure the same concept and link to the interrelationship of the items in the scale. The internal consistency of a scale should be determined before adopting it for research purposes, to ensure the validity of the data collected. An alpha value over 0.7 and 0.8 is considered to indicate good and high internal consistency, respectively. In the present study, the mean Cronbach’s alpha for each age group was considered to indicate consistency of responses in that group. The Cronbach’s alpha was computed for questions about smartphone usage level for the following 19 types of content: news, business, education, product/service, traffic, web document, game, adult content, movie/TV/video, music, web novel, sports betting, e-mail, messenger, SNS, product purchase, product selling, finance, and life management. Through this process, we found that each age group had a Cronbach’s alpha of over 0.8 for smartphone usage level for the 19 types of content.

### 2.2. Models

This study evaluates the smartphone addition level and its association with smartphone usage patterns and related expenditure. First, statistical hypothesis tests (t-test) are carried out to test whether there is a difference in smartphone usage patterns between the normal user group and the problematic user group. The usage and expenditure by content between the two groups are compared. In addition, by employing the data-driven prediction models based on machine learning techniques, the possibility of predicting smartphone addiction level by smartphone usage pattern is explored. The decision tree, random forest, and Xgboost algorithm are employed to solve the classification problem.

**Figure 1:**
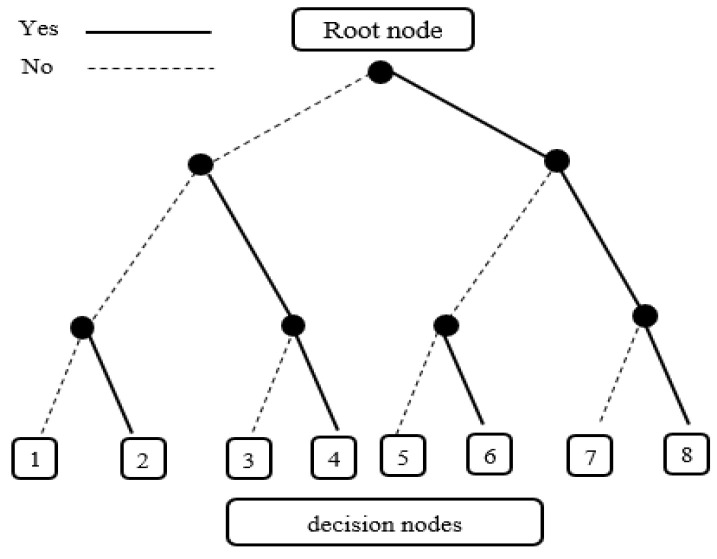
The decision Tree example.

Decision trees are non-parametric supervised learning method based on tree-like graph models, in which each branch represents a decision result on a predictor variable and its threshold [30]. Datasets are divided into smaller binary subsets, while associated decision trees are developed incrementally. To train trees, one predictor variable and one threshold are selected at a time to determine a branch at a node such that each branch has similar samples after the split. The tree grows in depth by adding one new node at a time. The result is a tree with a decision node and a leaf node based on imputed statistics and it can divide complex decisions into several simple decisions. Therefore, this model is effective for classifying addiction based on the pattern of the condition. The model has been employed in several studies to predict addictions [31,32]. An example of a decision tree has been presented in Figure 1.

If all the affected training instances belong to the same class of the decision tree, the node’s “Gini” index is equal to zero. The Gini index attribute on a node measures its impurity. The following equation shows how the training algorithm calculates the Gini score *Gi* of the *i*-th node.

(2) $$G_{i}=1-\sum_{k=1}^{n}p_{i,k}^{2},$$

where $p_{i,k}$ is the ratio of the training instance off the *i*-th node to the *k*-th class. While the Gini score is set as default, an impurity measure, the entropy score, is used as an alternative. Since the computation of the Gini score is faster, we set the Gini score as the default parameter.

(3) $$E\left(S\right)\;=\;\sum_{i=1}^{c}-p_{i}\log_{2}p_{i},$$

The random forest and Xgboost models are modified versions of the decision tree for classification. A random forest model is an ensemble of decision trees, generally trained via the bagging method [33]. The decision tree, however, is predisposed to overfitting; therefore, bagging, one of the ensemble methods, becomes necessary. Bagging provides better classification by averaging the results of similar overfitting models. The ensemble in the arbitrary decision tree is called the random forest model. The latter is appropriate for the present study because it would classify the smartphone addiction level based on a given set of variables. One of the advantages of random forest method is that there are few hyperparameters with the potential to strongly influence its performance. It is defined only by the number of trees and the depth of each tree. For a study using the random forest technique for addiction problems, please refer to [34,35].

Xgboost is a remodeling of the decision tree by the implementation of a gradient boost designed for speed and performance [36]. It is used for supervised learning problems, where training data with multiple features are used to predict a target variable. Based on the fundamental concepts used in the decision tree, the method of classifying features and predicting a target variable remains the same. Therefore, it is appropriate for the present study.

### 2.3. Measures and Procedure

Though the KISA data comprised 180 questions, to correspond with the subject of the study, 24 features of smartphone application usage and three general characteristics are selected. Note that the objective of this research is to predict smartphone addiction level information available from smartphones. The other variables are excluded since they cannot be obtained from the smartphone usage log data. As target variables, two different types of smartphone addiction levels were selected (Table 1).

The number of participants for each addiction type by age group is presented in Table 2.

The dataset is split into the training and test sets, by a ratio of 7:3. We then conducted the grid search process for training each of the three models (decision tree, random forest, Xgboost) to tune the hyper-parameters of the estimators. Any assigned parameter when constructing an estimator may be optimized in this manner. Table 3 shows which estimator was tuned in the assigned range of hyper-parameters for each of the three models.

A 5-fold cross validation method was performed for every model. Cross-validation is a resampling procedure used to evaluate machine learning models using a limited data sample. In k-fold cross-validation, the data are divided into number of k equally-sized segments or folds to identify the best performing training model.

## 3. Results

**Figure 2:**
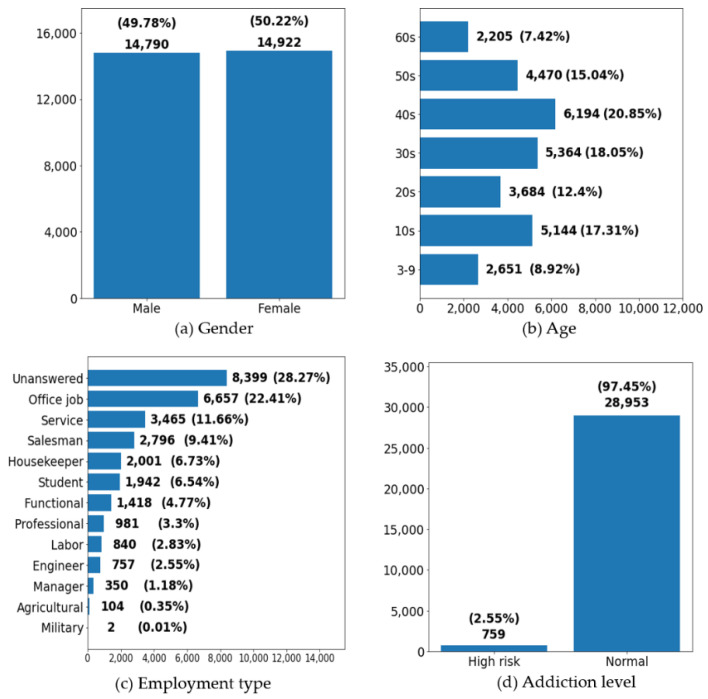
Users’ General Characteristics.

The basic statistics are presented in Figure 2.

Table 4 summarizes the smartphone usage level for each type of content based on addiction level. Except for office search, web document, and sports betting, significant differences were observed in the usage level of all content. The order of most used applications differed across addiction groups. Normal users responded that they used the messenger, news, SNS, and game applications most often, in that order, while problematic or high-risk users used messenger, game, music, and SNS applications, in that order.

Table 5 summarizes the monthly expenditure on smartphone use and frequency of usage. The normal and risk groups exhibited significant differences in the average monthly expenditure on games (t = −6.911, *p* < 0.001), movies/videos (t = −4.39, *p* < 0.001), and e-books and cartoons (t = −6.935, *p* < 0.001), and in the frequency of use on weekdays (t = −16.491, *p* < 0.001) and weekends (t = −16.707, *p* < 0.001). Specifically, those in the risk group spent 2, 1.64, and 2.4 times more money on games, movies/videos, and e-books and cartoons, respectively, as compared to those in the normal group. Further, they used their smartphone an average of 56.74 times on weekdays and 85.44 times on weekends. The risk group’s usage was 2.43 and 2.4 times higher on weekdays and weekends, respectively, as compared to those in the normal group.

Three predictive models are tested to forecast the smartphone addiction. Table 6 summarizes the accuracy rates of the models. The prediction accuracy for those in their 10s were the highest with random forest (86.36%), followed by Xgboost, with a small margin (84.45%). Both random forest and Xgboost exhibited an 80.48% accuracy rate for prediction among participants in their 20s. However, for those in their 30s, random forest exhibited the highest accuracy rate (80.95%), followed by Xgboost (77.38%). The decision tree model exhibited the least accuracy for all age groups. The average value for accuracy appeared in the following descending order for each age group: random forest, Xgboost, and decision tree. The average recall value for problematic and normal users was in the following descending order: decision tree, Xgboost, and random forest; random forest, Xgboost, and decision tree, respectively. Thus, the random forest and Xgboost models performed the best for every category. These levels of accuracy were accomplished using 27 variables that included 24 variables on smartphone usage and three pertaining to the participants’ personal information. Thus, the present findings suggest that data on the characteristics of smartphone usage by age and employment status of users are sufficient for determining whether an individual is addicted to a smartphone.

## 4. Discussion

In this research, the smartphone addition level and its association with smartphone usage patterns were studied. First, statistical t-tests were conducted to find out whether there is a difference in each type of content used by the normal user group and the problematic user group. We could figure out how different the usage patterns and expenditures are between the two groups. There were significant differences in the usage level of all contents except for the use of office search, web document and sports betting. Both groups were found to use messenger applications most frequently. However, there was a difference in the contents used in the second and third order. The normal use group responded that the second and third most frequently used applications were news and SNS, but the problematic user group responded that they used game and music apps as the second and third. These results are consistent with the results in [37], as it is stated that “the risk group for smartphone addiction played games more habitually and they did so for achieving targets on the game, as compared with the normal user group.” In addition, it is observed that there were significant differences in the average monthly expenditure on games, movies/videos, and e-books and cartoons between two groups. In particular, those in the problematic group spent 2, 1.64, and 2.4 times more money on games, movies/videos, and e-books and cartoons, respectively, as compared to those in the normal group. The results agree with the results in [38]. In [38], it is stated that addiction has the capacity to stimulate the users’ intention to purchase in-game apps.

Further, machine learning techniques were also used to predict the smartphone addiction level based on the usage pattern and the general characteristics of users. Three learning methods were considered and tested: random forest, Xgboost and decision tree. The average value for accuracy resulted in 82.59% (random forest), 80.77% (Xgboost) and 74.56% (decision tree), respectively. As evident from Table 7, the accuracy of the models did not differ significantly based on the presence and absence of users’ general characteristics such as gender and employment status. The average difference of the two conditions for each model was −4.05% for decision tree, −2.26% for random forest, and only −1.37% for Xgboost. The total average difference for the three models was only −2.56%. This suggests that the employment status and the gender of users do not contribute to the prediction of their smartphone addiction levels. The results are consistent with those of some research, which reported that smartphone addiction is not significantly related to gender [28,39,40]. To rule out the effect of multi-collinearity among these two categories (presence and absence of users’ general characteristics), we conducted an additional experiment to determine whether the above three demographic variables were reflected by the smartphone usage variables, by predicting the three variables based on the 24 variables pertaining to usage characteristics. The random forest model was applied in this process.

As evident from Table 8, based on the application usage information, the prediction accuracy for those in their 10s was the highest (91.1%), followed by that for those in their 20s (56.48%), and those in their 30s (48.21%). The average accuracy rate was 65.26%. This allowed us to conclude that the smartphone usage trends of teenagers are so unique that users’ application usage characteristics reflected their age features. Employment status was also predicted by random forest, when grouped by employment. The total average accuracy was the highest (85.08%), indicating that the users’ application usage patterns reflected their employment status. The prediction rate for average estimated sex was 56.76%, indicating that the gender of users could not be differentiated based on their application usage patterns. Indeed, according to a report by the Korea Information Agency, it has been proved that the gender itself does not affect smartphone usage [6]. These findings suggest that the sex variable is not necessary for estimating the smartphone addiction level. Further, these findings explain why the accuracy rates of models based on the presence or absence of users’ general characteristics did not differ significantly.

## 5. Conclusions

The objective of the present study was to explore the possibility of predicting smartphone addiction level with mobile phone log data. The 27 variables pertained to the users’ general characteristics and smartphone usage characteristics. The results were significant enough to deem the applicability of this method in practice. Further, we conducted an additional experiment to select an optimal model using lesser information by comparing the present results with those of predictive models that excluded users’ general characteristics. To our best knowledge, this is the first study to explore the possibility of predicting smartphone addiction level by smartphone usage pattern based on machine learning techniques.

The results show that information on users’ smartphone usage patterns and expenditure can be used as predictors to determine whether users are addicted to smartphones. Currently, existing questionnaires developed to diagnose smartphone addiction focus on the psychological factors of users. These surveys are obviously useful, but only when the user participates in the survey can the diagnosis of smartphone addiction be diagnosed. Since our machine learning based model can predict the level of smartphone addiction based on smartphone usage patterns alone, it can potentially be used to find risk groups for smartphone addiction. From a practical perspective, it is expected that it will be easier to develop a mobile app or program such as self-diagnostic applications because it is possible to predict only the smartphone usage pattern without personal information. In addition, since information on usage patterns provides more objective information, the results of this study can be used to improve existing surveys and to develop practical guidelines.

This study had some limitations. The data we used were based on the KISA survey. Though we verified the internal consistency among age groups by assessing the mean Cronbach’s alpha values, the data were not free from the limitations of the survey, specifically, the lack of objectivity. For instance, questions on the degree of use of a mobile application were based on the user’s perceived use, which may not be as accurate as actual objective usage.

Our studies can be extended in numerous directions. First, we can verify and extend our model using log data that capture the precise time spent on each smartphone application. Although it is difficult to obtain sufficient samples, it is expected that more specific information about frequently used apps, and the times when they are used for each type of content, can be obtained. In addition, data mining techniques can be applied to find various patterns of users related to smartphone addiction level. In [41], data mining techniques were applied to study the difference in perception and behavior of smartphone uses as well as the effect of addiction on learning. Similarly, techniques such as cluster analysis or association rule learning can be applied. Through cluster analysis, a group of smartphone users can be divided into several segments with similar behavioral characteristics, which will help to better understand the relationships between different groups of users. Additionally, the association rule can be employed to discover interesting relations between variables in our dataset.

## Figures and Tables

**Table 1:** Variables.

	Variables	Contents
Independent variables	● Age	
	● Sex	
	● Occupation	
	● Smartphone usage level by content	News
		Business
		Education
		Product/service Traffic
		Web document
		Game
		Adult content
		Movie/TV/video Music
		Web novel
		Sports betting
		E-mail
		Messenger
		SNS
		Product purchase
		Product selling
		Finance
		Life management
		Game
		Movie/TV/video
		e-book, web-toon, web-fiction
	● Monthly expenditure on each content	
	● Number of times of use on weekdays	
	● Number of times of use on weekends	
Target variables	Addiction type (1: High risk, 2: Normal)	

**Table 2:** The number of participants for each addiction type.

	Population	
	High Risk	Normal
10s	183	4961
20s	136	3548
30s	139	5225

**Table 3:** Grid Search Parameters.

Method	Hyper-Parameters		
Decision tree	Gini depth	Entropy depth	
	2, 3, 4, 5	2, 3, 4, 5	
Random forest	Sample split	Estimators	
	4, 5	100, 500	
Xgboost	Base score	Depth	Estimators
	0.5, 0.55, 0.6, 0.65, 0.7	2, 3, 4	10, 50, 70, 100

**Table 4:** Smartphone Usage Level for Each Usage Content.

19 Contents, 7-Point Likert Scale	Normal Group (*n* = 28,953)		Risk Group (*n* = 759)		t-Test	
	Mean	SD	Mean	SD	Normal vs. Risk Groups	
					t-Value	*p*-Value
News	3.34	2.480	4.18	1.917	−11.880	*p* < 0.001
Office search	2.44	2.385	2.25	2.555	2.096	*p* = 0.036
Education	1.47	2.116	2.29	2.549	−8.828	*p* < 0.001
Product/service search	2.44	2.366	3.58	1.919	−15.977	*p* < 0.001
Transportation	2.30	2.390	3.24	2.416	−10.545	*p* < 0.001
Web document	3.08	2.208	3.07	2.608	0.153	*p* = 0.879
Game	3.22	2.176	4.59	1.979	−18.714	*p* < 0.001
Adult content	0.28	1.018	0.53	1.354	−5.072	*p* < 0.001
Movie/TV/video	2.78	2.358	3.49	2.517	−7.748	*p* < 0.001
Music	2.99	2.467	4.20	2.306	−14.277	*p* < 0.001
Web novel	1.45	2.124	3.10	2.161	−20.819	*p* < 0.001
Sports betting	0.13	0.691	0.19	0.866	−1.819	*p* = 0.069
Email	1.61	2.157	2.82	2.366	−13.904	*p* < 0.001
Messenger	5.00	1.798	5.43	1.523	−7.650	*p* < 0.001
SNS	3.31	2.429	4.19	2.389	−10.076	*p* < 0.001
Product/service purchase	2.00	2.319	2.67	2.485	−7.312	*p* < 0.001
Product/service selling	0.56	1.431	1.08	1.959	−7.206	*p* < 0.001
Finance	1.74	2.290	2.20	2.495	−5.071	*p* < 0.001
Daily management	1.21	1.973	1.61	2.177	−5.108	*p* < 0.001

**Table 5:** Smartphone Usage Characteristics.

	Normal Group (*n* = 28,953)		Risk Group (*n* = 759)		t-Test	
	Mean	SD	Mean	SD	Normal vs. Risk	
					t-Value	*p*-Value
**Monthly expenditure on contents (won)**						
Game	1299.94	3815.277	2586.30	5090.486	−6.911	*p* < 0.001
Movie/TV/video	1032.54	3239.524	1694.33	4120.399	−4.390	*p* < 0.001
e-books/cartoons	487.93	1858.009	1181.82	2740.036	−6.935	*p* < 0.001
**Frequency of use (number)**						
Number of uses on weekdays	23.41	27.739	56.74	55.516	−16.491	*p* < 0.001
Number of uses on weekends	35.60	44.275	85.44	81.874	−16.707	*p* < 0.001

**Table 6:** Accuracy and Recall Value for Each Model.

Accuracy (%)			
	Decision tree	Random forest	Xgboost
10s	75.45	86.36	84.45
20s	76.82	80.48	80.48
30s	71.42	80.95	77.38
Average	74.56	82.59	80.77
Recall value			
	High recall	Normal recall	
Decision tree			
10s	0.80	0.71	
20s	0.84	0.68	
30s	0.79	0.64	
Average	0.81	0.67	
Random forest			
10s	0.83	0.89	
20s	0.77	0.84	
30s	0.81	0.81	
Average	0.80	0.85	
Xgboost			
10s	0.83	0.86	
20s	0.77	0.84	
30s	0.83	0.71	
Average	0.81	0.80	

**Table 7:** Accuracy Difference.

Accuracy (%)			
	Decision tree		
	Usage and personal information	Usage only	Difference
10s	75.45	68.18	−7.27
20s	76.82	71.95	−4.87
30s	71.42	71.42	0.00
Average	74.56	70.51	−4.05
	Random forest		
	Usage and personal information	Usage only	Difference
10s	86.36	84.45	−1.91
20s	80.48	79.26	−1.22
30s	80.95	77.28	−3.67
Average	82.59	80.33	−2.26
	Xgboost		
	Usage and personal information	Usage only	Difference
10s	84.45	82.72	−1.73
20s	80.48	80.48	0.00
30s	77.38	75.00	−2.38
Average	80.77	79.40	−1.37

**Table 8:** Prediction based on Users’ General Characteristics.

Prediction Based on Users’ General Characteristics	Accuracy (%)
Based on age group	
10s	91.10
20s	56.48
30s	48.21
Average	65.26
Based on employment status (whether employed)	
No	72.87
Yes	93.75
Average	85.08
Based on sex	
Male	57.34
Female	56.12
Average	56.73

## Data Availability

Restrictions apply to the availability of these data. Data was obtained from Korea Internet & Security Agency and are available with the permission of Korea Internet & Security Agency.
